# Intratumoral Decorin Gene Delivery by AAV Vector Inhibits Brain Glioblastomas and Prolongs Survival of Animals by Inducing Cell Differentiation

**DOI:** 10.3390/ijms15034393

**Published:** 2014-03-12

**Authors:** Hsin-I Ma, Dueng-Yuan Hueng, Hao-Ai Shui, Jun-Ming Han, Chi-Hsien Wang, Ying-Hsiu Lai, Shi-Yuan Cheng, Xiao Xiao, Ming-Teh Chen, Yi-Ping Yang

**Affiliations:** 1 Department of Neurological Surgery, Tri-Service General Hospital, National Defense Medical Center, Taipei 11490, Taiwan; E-Mails: uf004693@mail2000.com.tw (H.-I.M.); hondy2195@yahoo.com.tw (D.-Y.H.); d49519001@ym.edu.tw (J.-M.H.); ssfwang@yahoo.com.tw (C.-H.W.); 2 Graduate Institute of Medical Sciences, National Defense Medical Center, Taipei 11490, Taiwan; E-Mail: haoai@ndmctsgh.edu.tw; 3 Department of Biotechnology and Laboratory Science in Medicine, National Yang-Ming University, Taipei 11221, Taiwan; 4 Department of Medical Research and Education, Taipei Veterans General Hospital, Taipei 11217, Taiwan; E-Mail: d49405004@gmail.com; 5 Department of Neurology, Northwestern Brain Tumor Institute, The Robert H. Lurie Comprehensive Cancer Center, Center of Genetic Medicine, Northwestern University Feinberg School of Medicine, Chicago, IL 60611, USA; E-Mail: shiyuan.cheng@northwestern.edu; 6 Division of Molecular Pharmaceutics, Eshelman School of Pharmacy, University of North Carolina at Chapel Hill, Chapel Hill, NC 27599, USA; E-Mail: xxiao@email.unc.edu; 7 School of Medicine, National Yang-Ming University, Taipei 11221, Taiwan; 8 Department of Neurosurgery, Neurological Institute, Taipei Veterans General Hospital, Taipei 11217, Taiwan; 9 Institute of Clinical Medicine, National Yang-Ming University, Taipei 11221, Taiwan

**Keywords:** dsAAV, decorin, glioblastoma multiforme, proteomics, 2-D electrophoresis

## Abstract

Glioblastoma multiforme (GBM) is the most malignant cancer in the central nervous system with poor clinical prognosis. In this study, we investigated the therapeutic effect of an anti-cancer protein, decorin, by delivering it into a xenograft U87MG glioma tumor in the brain of nude mice through an adeno-associated viral (*AAV2*) gene delivery system. Decorin expression from the AAV vector *in vitro* inhibited cultured U87MG cell growth by induction of cell differentiation. Intracranial injection of AAV-decorin vector to the glioma-bearing nude mice *in vivo* significantly suppressed brain tumor growth and prolonged survival when compared to control non-treated mice bearing the same U87MG tumors. Proteomics analysis on protein expression profiles in the U87MG glioma cells after AAV-mediated decorin gene transfer revealed up- and down-regulation of important proteins. Differentially expressed proteins between control and AAV-decorin-transduced cells were identified through MALDI-TOF MS and database mining. We found that a number of important proteins that are involved in apoptosis, transcription, chemotherapy resistance, mitosis, and fatty acid metabolism have been altered as a result of decorin overexpression. These findings offer valuable insight into the mechanisms of the anti-glioblastoma effects of decorin. In addition, AAV-mediated decorin gene delivery warrants further investigation as a potential therapeutic approach for brain tumors.

## 1. Introduction

Glioblastoma multiforme (GBM) is classified as WHO grade 4 astrocytic glioma and is the most common and aggressive type of primary brain tumor [1–3]. Although chemotherapy, radiotherapy and surgery have been used to treat GBM in the clinic, the median survival time of patients with gliomas is only 13 to 16 months [4]. Gene therapy is an alternative and promising approach for the treatment of various types of human cancers including malignant brain tumors [5,6]. Various viral vectors have been designed to deliver therapeutic genes into cancer cells [7]. A number of gene therapy strategies have been approved for clinical trials testing their efficacies in patients with different types of cancers. For glioma gene therapy, oncolytic viruses as well as viral vectors that contain suicidal prodrug converting genes, such as thymidine kinase and cytosine deaminase genes, have been investigated [5]. We previously showed that vectors based on adeno-associated virus serotype 2 (*AAV2*) could be used as an effective vector for delivering therapeutic genes to the tumor and effectively suppressed growth of malignant gliomas in the brain of mice [5,8].

AAV is a promising tool for gene therapy of human diseases [9,10]. AAV mediates long-term gene expression with no apparent toxicity [11–13]. The lack of pathogenicity and cytotoxicity to normal cells renders the AAV vector a suitable candidate for treating human gliomas in the clinic. Recently improvement of AAV vectors that contain DNA genomes in the double-stranded (dsAAV) hairpin form (also termed self-complementary scAAV) offers more rapid and robust gene expression both *in vitro* and *in vivo* [12,14,15]. However, studies that employ dsAAV as a tool for evaluation of therapeutic effects on glioma growth in the brain of animals and protein expression have not been reported.

Decorin is a small leucine-rich proteoglycan that is involved in multiple cellular processes. In addition to its roles of modulating matrix assembly, fibrogenesis and cell proliferation [16], decorin displays anti-cancer activities through affecting signaling pathways of epidermal growth factor receptor (EGFR), transforming growth factor-beta (TGF-beta), and p21 [17]. Decorin directly binds and activates EGFR as an EGFR ligand and causes a down-regulation of EGFR and its signaling, leading to a growth inhibition and cell differentiation in certain non-glioma cancer cells [18,19]. Additionally, decorin also causes cell growth arrest by suppressing extracellular activities of TGF-beta, and inducing the expression of p21 in cells [17]. TGF-beta is a prominent glioblastoma-associated immunosuppressant that mediates escape of glioma cells from immune surveillance. TGF-beta not only interferes with multiple steps of afferent and efferent immune responses, but also stimulates cell migration, invasion and tumor angiogenesis [20]. Previous studies using an adenoviral vector showed that expression of decorin suppresses TGF-beta signaling [21–24]. However, the effects of decorin expression on glioma cells, including cell differentiation and proliferation have not been examined. Since decorin has been shown to have a differentiation effect on other types of human cancer cells [18,19], we hypothesized that decorin also has a differentiation effect on glioblastoma cells.

Proteomics is a new technology that simultaneously studies the expression of a vast majority of cellular proteins, the proteome, (rather than individual proteins) in a cell, tissue, or an organ under various experimental conditions. Proteomic analysis of global proteins in a cell can provide better insight into the underlying mechanisms of therapeutic treatments such as gene delivery by a viral vector as well as its therapeutic potentials [25,26]. Additionally, proteomics has been employed to study differentiation effects resulting from decorin expression in breast cancer cells [18]. Moreover, different types of human cancer cells could have different responses to decorin treatment, and different viral vector delivery methods could also lead to different proteome alterations. To test the possibility of glioma gene therapy using AAV vector expressing decorin, we performed both *in vivo* and *in vitro* experiments, inhibiting human glioma growth in the brain of nude mice and proteomic analysis on cultured U87MG glioblastoma cells. Our results show that decorin gene transfer effectively suppressed U87MG brain glioma growth in mice and induced a differentiation phenotype of U87MG cells with modulation of various proteins that are critical in cell differentiation, proliferation, survival and metabolism.

## 2. Results and Discussion

### 2.1. Induction of U87MG Cell Differentiation by dsAAV CMV-Decorin Vector

**Figure 1.**
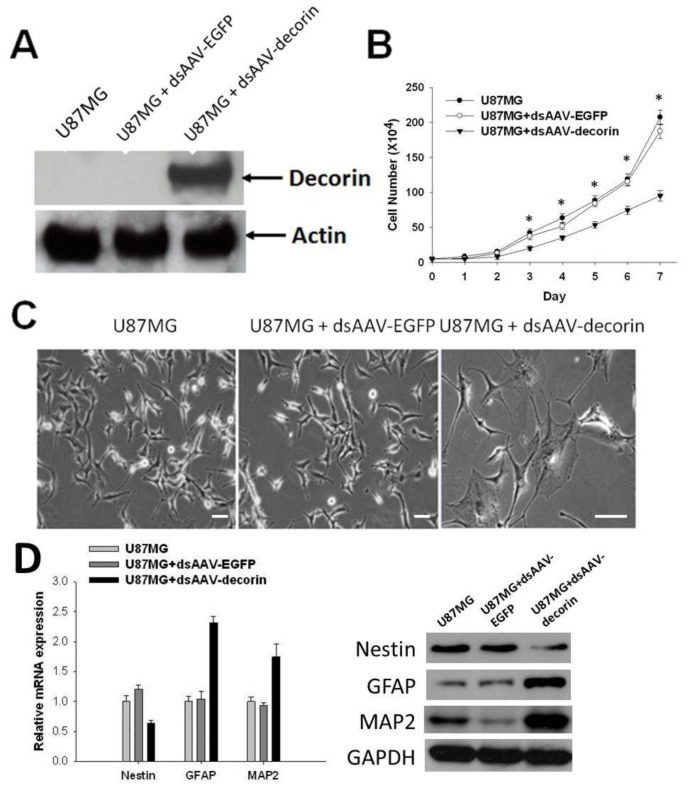
Decorin expression inhibits cells proliferation and induces changes of morphology of U87MG cells. (**A**) Western blot analysis of expression of dsAAV-encoded decroin in U87MG cells. No endogenous decorin proteins were detected in mock-transduced (U87MG) or dsAAV-EGFP cells while high-levels of ectopic decorin was seen in U87MG-dsAAV-decorin cells. Beta-actin was used as a loading control; (**B**) Inhibition of cell proliferation in dsAAV-decorin-transduced U87MG cells. The cell numbers of U87MG, stably transduced U87MG-dsAAV-EGFP and decorin were evaluated at the indicated times. Decorin expression suppresses cell growth in U87MG-dsAAV-decorin cells compared to Mock- or EGFP-transduced U87MG cells; (**C**) Decorin induced significant changes of morphology of U87MG-dsAAV-decorin cells as compared to controls; (**D**) mRNA and protein expression levels of Nestin, GFAP and MAP2 were detected in U87MG, U87MG-dsAAV-EGFP and decorin cells. Data are representative of three independent experiments. * *p* < 0.05; Scale bar: 20 μm.

To evaluate the effects of decorin expression in U87MG glioma growth and cell proliferation, we established U87MG cells that stably express dsAAV-encoded decorin or EGFP control. As shown in Figure 1A, in mock-infected U87MG cells or U87MG dsAAV-EGFP cells, no decorin proteins were detected. However, in U87MG-AAV-decorin cells, high-levels of decorin expression were detected. Next, we examined the impact of AAV-mediated decorin gene expression on U87MG cell proliferation and differentiation *in vitro*. As shown in Figure 1B, the proliferation rate of U87MG cells in dsAAV-decorin group was significantly slower than those in either the mock infected U87MG or dsAAV-EGFP infected control groups (*p* < 0.01), whereas proliferation rate between the latter two control groups (U87MG and U87MG + dsAAV-EGFP) were similar. These results indicate that decorin expression suppressed cell proliferation of U87MG cells and that overexpression of control EGFP proteins by AAV did not affect the growth of the cells, excluding non-specific effects from either the virions or levels of non-specific protein expression.

To determine whether the retarded growth of U87MG cells is caused by decorin-induced cell differentiation, we examined the morphology as well as the protein and mRNA expression levels of Nestin, GFAP and MAP2 of various U87MG cells. As shown in Figure 1C, treatment of U87MG cells with dsAAV-decorin induced significant morphological change in the cells, showing more extended and flattened morphology as compared to EGFP-transduced and non-transduced cells (U87MG + dsAAV-decorin *vs.* U87MG + dsAAV-EGFP and U87MG). The mRNA and protein levels of Nestin, GFAP and MAP2, which are markers of neural progenitor cells, glial cells and neurons, respectively, showed a pro-differentiation tendency of the AAV-decorin treated cells in comparison to the non-treated cells (Figure 1D). This data support a pro-differentiation tendency in the AAV-decorin treated cells.

### 2.2. Long-Term Survival of Glioma-Bearing Nude Mice Treated with AAV-Decorin

**Figure 2.**
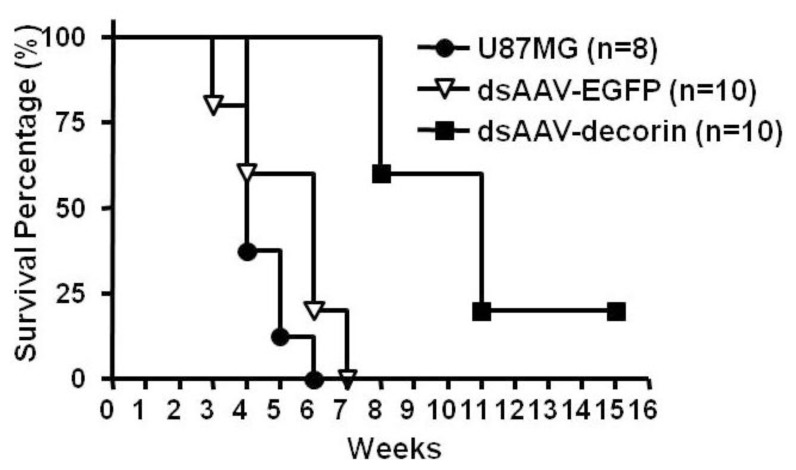
Gene delivery of dsAAV-decorin prolonged the survival of nude mice that bear malignant U87MG gliomas in the brain. U87MG cells (5 × 10^5^/mouse) were stereotactically injected (i.c.) into the brain of nude mice. Five days post-implantation, dsAAV-decorin vectors were delivered at MOI = 10,000 via intraturmal injection through the same barrel hole in the skull where the tumor cells were injected. No mice died until the 4th week after tumor transplantation. Because of excessive tumor growth, all mice without dsAAV-decorin treatment (*n* = 8) died within six weeks. The mice in the dsAAV-EGFP group (*n* = 10) all died within seven weeks. However, mice that received dsAAV-decorin treatment (*n* = 10) were alive at the 6th week. At the 11th week and forward, ~20% of those mice survived for a much longer period of time. The experiments were performed two independent times with similar results (*p* < 0.0001).

In the first part of this *in vivo* study (the therapeutic group), 4-week-old nude mice were intracranially implanted with the glioma U87MG cells. Five days post-implantation, dsAAV-decorin vectors were injected intratumorally in the brain through the original injection site used for U87MG implant. After 5~6 weeks, mice that received control U87MG tumors developed neurological symptoms due to the tumor burden in the brain and died within six weeks post-implantation (Figure 2). In contrast, mice that received the U87MG implant and also the subsequent dsAAV-decorin injection (multiplicity of infection (MOI) = 10,000) showed a significantly improved survival rate. Mice did not die until the 8th week after tumor implantation. At the 11th week, 20% of mice with U87MG-AAV-decorin tumors were still alive and they survived longer than 14 weeks after the initial intracranial U87MG tumor implantation.

**Figure 3.**
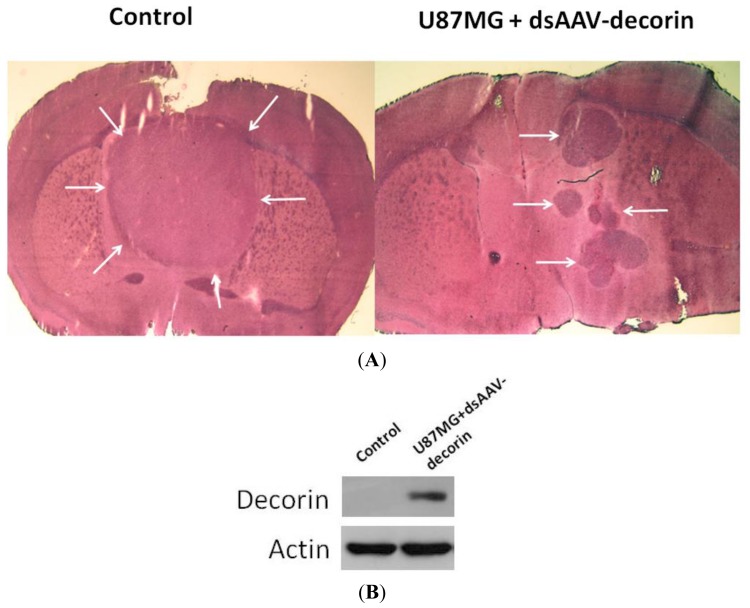
dsAAV-Encoded decorin significantly inhibited the growth of U87MG glioma in the brain of mice. (**A**) Several mice in U87MG-dsAAV-decorin group and all control mice shown in Figure 2 were euthanized at six weeks post-implantation. Brains of these mice were removed, sectioned and analyzed by H&E staining. The mice in the untreated control group had formed large, solid tumors in the brain with an average volume of 48.3 ± 4.5 mm^3^. On the contrary, in the treated group (dsAAV-decorin), the growth of the U87MG cells was significantly inhibited and formed small tumors as separate nodules in the brain. The data are representative of two independent experiments; (**B**) The tumor specimens from each group were dissected and analyzed by Western Blot to confirm the existence of decorin in dsAAV-decorin treated tumors. White arrows indicate tumor nodules in the brain.

As shown in Figure 3A, six weeks post-implantation, mice that received U87MG-dsAAV-EGFP cells developed large brain tumors with an average volume of 48.1 ± 4.3 mm^3^ whereas surviving mice that received U87MG-dsAAV-decorin cells had much smaller tumors in separate nodules with a smaller average volume of 15.2 ± 3.2 mm^3^ in the brain. Western blot analysis confirmed the existence of decorin in the dsAAV-decorin treated tumors (Figure 3B). This data shows that expression of dsAAV-encoded decorin by U87MG gliomas significantly inhibited tumor growth in the brain of mice. In a separate set of experiments, we assess the impact of preventive treatment of U87MG tumors by co-injection of dsAAV-decorin viral particals with U87MG cells into the brain of mice.

**Figure 4.**
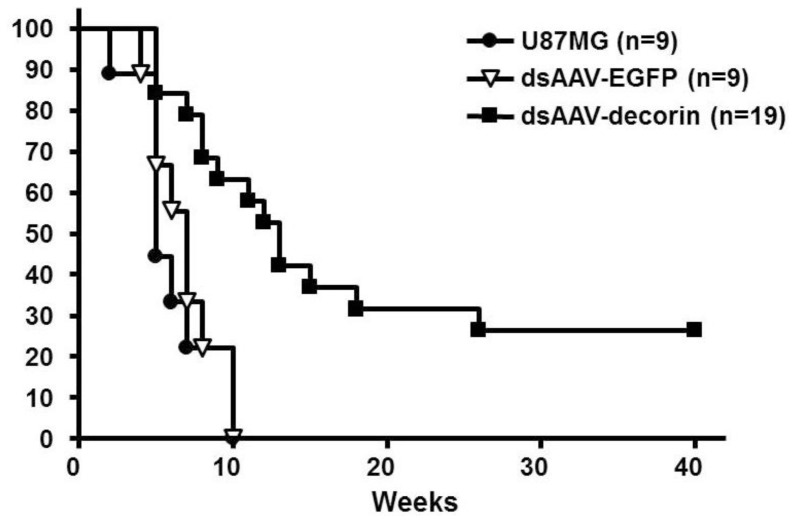
Co-delivery of dsAAV-decorin viral particles with U87MG cells results in a long-term survival of the nude mice that received U87MG cells. U87MG cells (5 × 10^5^) alone or tumor cell mixed with dsAAV-decorin vectors were co-injected i.c. into the brain of nude mice. Among all groups, none of the mice died until three weeks after tumor cell implantation. Afterwards, mice that received U87MG cells (*n* = 9) and dsAAV-EGFP (*n* = 9) gradually died within 10 weeks because of excessive tumor growth. However, over 50% of the mice that received dsAAV-decorin viral particles (*n* = 19) had survived at 10 weeks and 20% of the mice surviving for long period. The data are representative for two independent experiments (*p* < 0.001).

We then analyzed the prevention efficacy of dsAAV-decorin in brain tumors. As shown in Figure 4, in control groups of mice that received U87MG cells with mock or dsAAV-EGFP, all mice died within 10 weeks after tumor cell transplantation. In contrast, mice that received a mixture of U87MG tumor cells and dsAAV-decorin particles died gradually starting at the 8th week with a much slower pace. On the 18th week post-implantation, 22% mice were alive and from the 26th week on, 20% of treated mice survived until the experiment was terminated, one year post-implantation. No tumor or neo-lesions were found in the brains of these (20% surviving group) mice (data not shown).

### 2.3. Proteome Profiles of 2-DE in U87MG Cells from U87MG, U87MG + AAV-Decorin and U87MG + AAV-EGFP Groups

**Figure 5.**
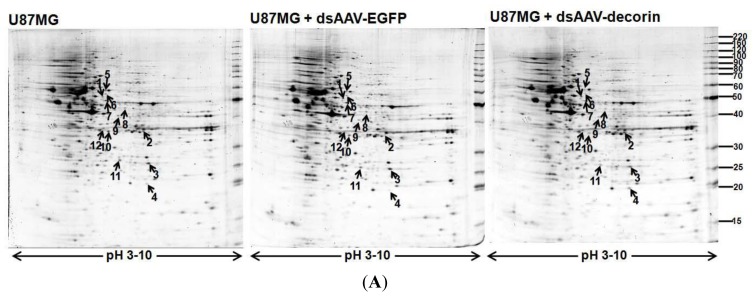

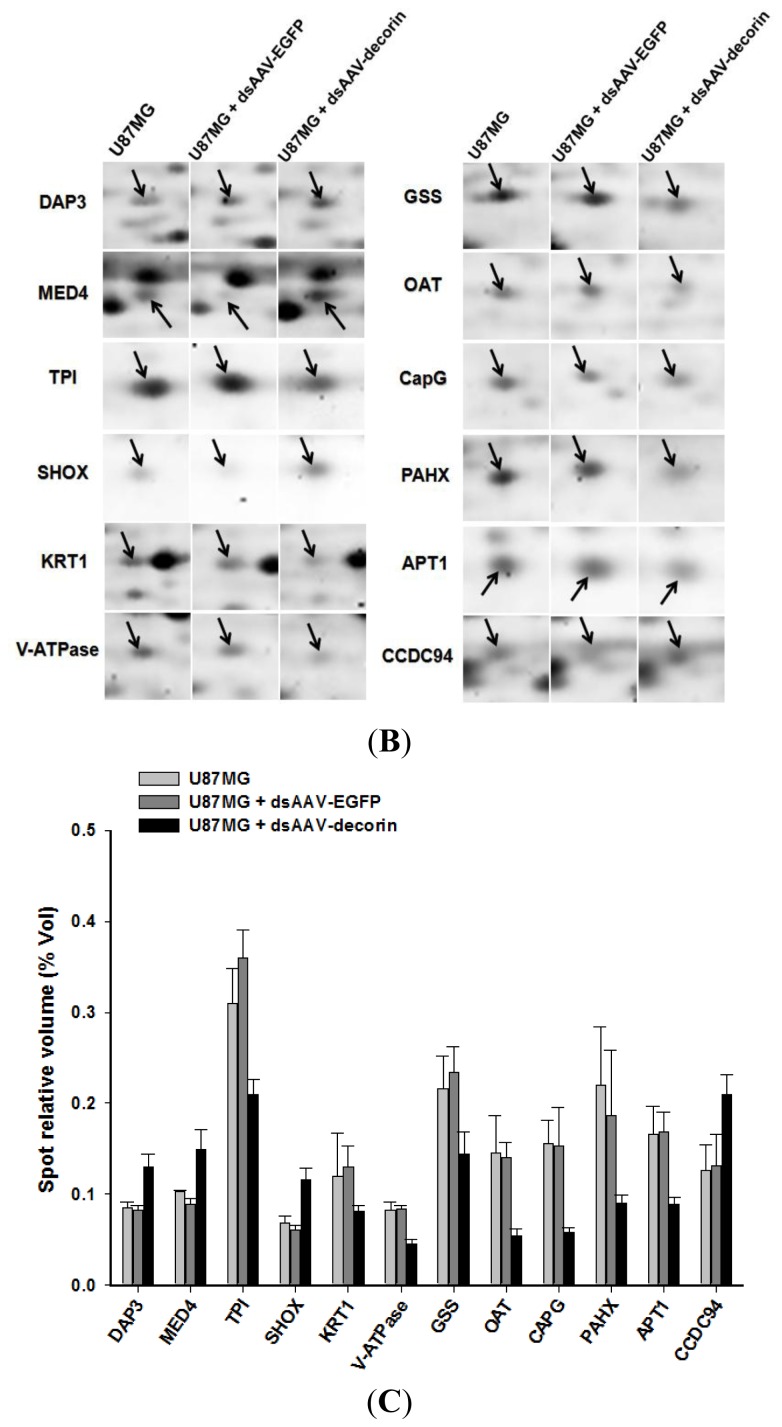
2-DE gels analysis and quantification of the protein content in U87MG, U87MG + dsAAV-EGFP, and U87MG + dsAAV-decorin cells. (**A**) The differentially expressed proteins between U87MG + dsAAV-decorin and U87MG + dsAAV-EGFP are indicated with arrows and labeled with numbers that are the same as those in Table 1. The p*I* range is shown at the bottom of the gels, while the molecular mass is indicated on the right side. The data are representative for three independent experiments; (**B**) The magnified images of protein spots that are either up- or down-regulated in U87MG cells by dsAAV-decorin transduction are individually displayed; (**C**) Statistical data of quantity of individual proteins are shown. All differences between U87MG + dsAAV-decorin and U87MG + dsAAV-EGFP groups, as well as between U87MG + dsAAV-decorin and U87MG groups were significant with *p* < 0.05. The data are representative for three independent experiments. Black arrows indicate the target proteins listed in Table 1.

In order to understand the underlying molecular changes in U87MG glioma cells induced by dsAAV-decorin transduction, a 2-DE-based proteomic analyses for the U87MG cells of different groups were performed. As shown in Figure 5A, protein spot patterns of 2-DE gels for cells from U87MG, U87MG + dsAAV-decorin and U87MG + dsAAV-EGFP groups were similar, but there were significant differences in the expression of individual proteins.

Although transduction of dsAAV-EGFP did not induce cell differentiation of U87MG cells, we observed that expression of several proteins was changed by dsAAV-EGFP, indicating that virion or protein load actually affected expression of these proteins (data not shown). We therefore used the U87MG + dsAAV-EGFP group as control to evaluate the impact of decorin expression on proteomes of U87MG cells to rule out the non-specific effects. As shown in Figure 5A, a total of 12 proteins (indicated by the arrows) were significantly up- or down-regulated by transduction of dsAAV-decorin as compared to the transduction of dsAAV-EGFP (U87MG + dsAAV-decorin *vs.* U87MG + dsAAV-EGFP). In contrast, no difference in expression of these proteins between mocked transduction and transductions of dsAAV-EGFP (U87MG *vs.* U87MG + dsAAV-EGFP) was found. Afterwards, these proteins were identified by peptide mass fingerprint (PMF). The characteristics of the identified proteins are listed in Table 1. As shown in Figure 5, magnified spots show images of the identified proteins (Figure 5B) and the relative intensity (percentage spot volume) of each protein spot (Figure 5C). These proteins are known to play critical roles in cell apoptosis, cell mitosis, gene transcription, chemotherapy resistance of cancer cells, and fatty acid metabolism and modification [27–31].

### 2.4. Identification of *AAV-Decorin*-Regulated Proteins Using Peptide Mass Fingerprint (PMF)

#### 2.4.1. Proteins as Apoptosis Mediator

Death-associated protein 3 (DAP3; spot 1, Table 1), is a novel positive mediator of apoptosis involved in death of cancer cells [32,33]. Its expression was up-regulated in U87MG + dsAAV-decorin cells (Figure 5B,C).

#### 2.4.2. Proteins for Transcription Regulation

**Figure 6.**
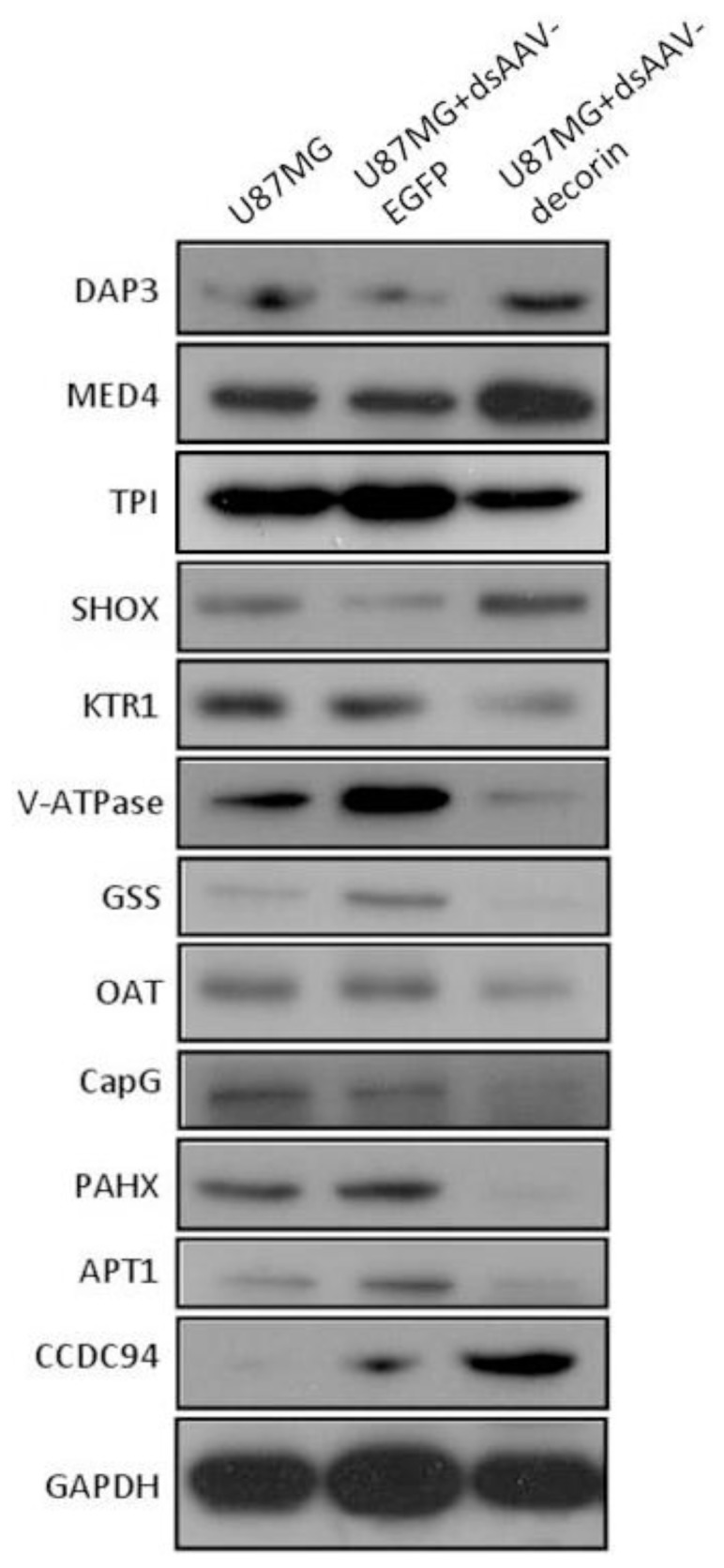
Multi-connections of decorin with critical biochemical mechanisms to GBM biology. Western blot showing the protein expression patterns that are up- or down-regulated in U87MG cells by dsAAV-decorin transduction.

Mediator of RNA polymerase II (MED4; spot 2, Table 1), an important coactivator of regulatory transcriptional factors responsible for cell development and differentiation [30], was up-regulated in dsAAV-decorin treated U87MG cells (Figure 6B,C). Short statue homeobox (SHOX; spot 4, Table 1) is a transcriptional protein existing in a wide variety of tissues, and shown to play roles in organ development during embryogenesis [34,35]. Its expression was also up-regulated in dsAAV-decorin treated U87MG cells (Figure 6B,C).

#### 2.4.3. Proteins Responsible for Chemotherapy Resistance

V-type proton ATPase (V-ATPase; spot 6, Table 1), a key role in the acidification of the tumor microenvironment [28], and glutathione synthetase (GSS; spot 7, Table 1), an enzyme catalyzing glutathione synthesis [36], were all down-regulated in dsAAV-decorin treated U87MG cells.

#### 2.4.4. Proteins Regulating Cytoskeleton Organization and Mitosis

Ornithine aminotransferase (OAT; spot 8, Table 1), an old enzyme with a newly discovered role in mitosis [37], was down-regulated in the dsAAV-decorin treated cells. Macrophage-capping protein (CAPG; spot 9, Table 1), a member of gelsolin superfamily proteins that control actin organization [38], was down-regulated in dsAAV-decorin treated U87MG cells.

#### 2.4.5. Proteins Involved in Fatty-Acid Metabolism and Fatty-Acid Modification of Protein

Acyl protein thioesterase (APT), an enzyme responsible for release palmitate (a 16-carbon saturated fatty acid) from a palmitoylated protein [27], and Phytanoyl-CoA hydroxylase (PAHX) an enzyme catalyzing the α-oxidation of phytanic acid [39,40] were also down-regulated in dsAAV-decorin treated U87MG cells.

### 2.5. Multi-Connections of Decorin with Various Biochemical Mechanisms Critical to GBM Biology

We further confirmed the protein expression patterns that are up- or down-regulated in U87MG cells by dsAAV-decorin transduction by Western blot analysis. DsAAV-decorin transduction increased the expression of DAP3, MED4, TPI, SHOX, CCDC94, and decreased the expression of KTR1, V-ATPase; GSS, OAT, CAPG, PAHX, APT1 (Figure 6), which was in accordance with the observations in magnified spot images of the identified proteins (please refer to Figure 5). In addition, we used a literature-based network analysis of all MEDLINE records and the Cytoscape open-source bioinformatics software platform to group the target linkage from our proteome profile data for a bioinformatics analysis illustrating multiple connections of decorin with various biochemical mechanisms critical to GBM biology (data not shown).

### 2.6. Discussion

Although decorin has been known to have an effect on cell differentiation of several types of human cancer cells [18,19], it remains unknown whether decorin has a similar effect on human glioma cells. Our present study demonstrates that treatment of malignant human glioma tumors with dsAAV-encoded decorin significantly inhibits growth of brain gliomas in animals, and transduction of dsAAV-decorin markedly suppress glioma cell proliferation, and shifts the morphology, as well as marker expression of U87MG glioblastoma cells, to a more differentiated state. It is well known that decorin is a potential therapeutic cargo to be delivered into glioblastoma cells for gene therapy, as demonstrated by an animal study showing that delivery of the decorin gene into glioblastoma cells can reduce tumor size [21–24]. Our data suggests that in addition to the effects of decorin described in previous studies on suppression of TGF-beta signaling [21–24], the anti-cancer effect of decorin is also attributed to the impact of decorin expression on cell differentiation of cancer cells.

In our therapeutic treatments using dsAAV-decorin for inhibition of intracranial U87MG gliomas, we used two separate approaches, a therapeutic inhibition and a preventive treatment. It is interesting that the overall survival rate of treated mice and prolonged mouse survival were similar in both of treated groups. The most plausible reason for this observation is that the expression of dsAAV is much faster than the single-stranded AAV [41]. Importantly, decorin is a secreted protein and acts on EGFR at the surface of glioma cells. Thus, dsAAV-encoded decorin could be secreted from the infected glioma cells, diffuse to many of the nearby implanted glioma cells, then inhibit their growth by attenuating EGFR and its signaling without the necessity of re-infecting those glioma cells. That could explain why intratumoral injection is just as effective as the *in vitro* infection in the prevention group. Therefore, this data has a significant clinical implication in designing gene therapy for glioma treatment.

Apoptosis is an important mechanism underlying death of cancer cells induced by various factors, such as chemotherapy drugs. DAP-3 (spot 1, Table 1) is a proapoptotic protein newly discovered from the screening of novel death genes in cancer cells. DAP-3 is ubiquitously expressed in different cells and tissues, and functions as a major positive mediator of cell death [32]. Transfection with a vector expressing antisense DAP-3 RNA caused a significant increase in the fraction of cells that remained viable in the continuous presence of interferon alpha [32], while overexpression of DAP-3 from a constitutive promoter induced the death of a variety of cell lines and primary cell cultures [29]. DAP3 is a critical target molecule for the treatment of osteosarcomas [42], and its apoptotic and tumor suppressive functions could be useful for cancer therapy [33]. Our proteomic data revealed that decorin up-regulated DAP-3 expression in U87MG glioma cells, suggesting that decorin could evoke an apoptotic mechanism through DAP3 to promote glioma cell death.

MED4 (spot 2, Table 1), a member of the mediator complex, which comprises approximately 20 different proteins, is an important coactivator of transcriptional factors responsible for cell development and differentiation [30]. Together with the up-regulation of two transcriptional factors, SHOX (spot 4, Table 1) and CCDC94 (spot 12, Table 1), decorin could directly regulate gene expression at the transcription level in glioma cells.

In various human cancers, genes of the glycolysis pathway have been found to be over-expressed, consisting with the theory that most cancer cells can tolerate low oxygen by using anaerobic glycolysis metabolism [43]. Triosephosphate isomerase (TPI) (spot 3, Table 1), which catalyzes the reversible interconversion of the triose phosphate isomers, dihydroxyacetone phosphate and d-glyceraldehyde 3-phosphate, is one of the glycolysis enzymes that improves drug resistance and survival of cancer cells [44]. TPI up-regulation has been found in melanoma, pancreatic and colon cancer [45–47]; TPI overexpression correlates well with the enhanced invasion ability of cancer [47]. Our results demonstrate that decorin suppresses the expression of TPI, possibly increasing the vulnerability of human glioma cells in an anaerobic environment.

V-ATPase (spot 6, Table 1) is a family of multi-subunit ATP-dependent proton pumps involved in a wide variety of physiological processes [48]. V-ATPase plays a key role in the acidification of the tumor microenvironment, and contributes to the progression of cancer from benign to malignant growth, and has a role in resistance to chemotherapy by impairing the uptake of weakly basic chemotherapeutic drugs and reducing their effect on tumor proliferation and metastatic behavior [28,49]. Several types of human cancer cells including glioma cells [50], are characterized by an increased V-ATPase expression and activity, and pretreatment with V-ATPase inhibitors sensitizes tumor cell lines to treatment by a variety of anti-cancer drugs [28]. Glutathione synthetase (GSS) (spot 7, Table 1) is an enzyme catalyzing the last step of glutathione [36]. Glutathione, one of the most important antioxidants in the eukaryotic organism [36], also contributes to resistance of cancer cells to chemotherapy, and plays a role in the inhibition of cytotoxicity induced by chemotherapeutic drugs by glutathione-mediated detoxification of compounds [51]. The level of glutathione predicts drug resistance in head and neck cancer, mammary carcinoma and malignant glioma cells [52,53]. Inhibition of glutathione synthesis or storage in tumors is a practical anti-cancer strategy [31]. Thus, the down-regulation of V-ATPase and GSS in U87MG + dsAAV-decorin cells suggests a decrease in chemo-resistance in glioma cells.

Ornithine aminotransferase (OAT) (spot 8, Table 1), an enzyme originally known to participate in mitochondrial arginine synthesis, was recently discovered to have a novel role in control mitosis of cells [37]. AB-5, an alkaloid drug, blocks cancer cell division through binding to OAT instead of tubulin [54], and thus has low chemotherapeutic toxicity as compared to traditional tubulin-binding antimitotics [55]. OAT expression can be stimulated by androgens, and is associated with the severity of prostate cancer [56]. The down-regulation of OAT by decorin suggests a reduction in mitosis and slower cell proliferation in U87MG-AAV-decorin cells.

CAPG (spot 9, Table 1) is a member of the gelsolin family of actin-regulatory proteins. Gelsolin superfamily proteins are calcium-sensitive proteins that control actin organization by severing filaments, capping filament ends and nucleating actin assembly [38]. CAPG is also a putative oncoprotein, as it is expressed at higher levels in metastasizing cancers, and its nuclear localization correlates well with invasive ability of cancer cells [57,58]. In addition, CAPG has an anti-apoptosis role, and plays important roles in promoting motility of both normal and cancer cells [59]. Therefore, down-regulation of CAPG by dsAAV-decorin could counteract the invasion ability and increase the apoptosis of GBM cells.

Modification of proteins by palmitate, a 16-carbon saturated fatty acid, can influence membrane binding and membrane targeting of modified proteins, and affect signal transduction. Just like phosphorylation and dephosphorylation of proteins, cycles of palmitoylation and depalmitoylation allow change of protein activities. Many oncoproteins, such as Ras, Hedgehog, Wingless use palmitoylation to regulate the extent of long- or short-range signaling [60]. Aberrant expression of enzymes responsible for palmitoylation has been shown to be a characteristic in cancer tissue [27]. In addition, palmitoylation has been shown to play a role in the growth of breast cancer cells [61], and apoptosis of cervical cancer cells [62]. Therefore, the decorin-induced down-regulation of APT, a key enzyme for depalmitoylation, may affect the survival and growth of human glioma cells [27].

## 3. Materials and Methods

### 3.1. Construction and Production of dsAAV2 Vector

The original dsAAV-CMV-EGFP shuttle vector has been described previously [41,63]. The plasmid dsAAV-CMV-decorin was constructed by replacing the *GFP* gene of dsAAV-CMV-EGFP with the mouse decorin cDNA at *Bam*HI and *Not*I sites [41]. The recombinant viral stocks were produced by the adenovirus-free, triple-plasmid co-transfection method [64]. The AAV vectors were purified by double CsCl density centrifugation, and the titers were determined by dot blot assay in the range of 5 × 10^12^ to 1 × 10^13^ viral particles per milliliter. All AAV vectors used in this study were derived from AAV serotype 2.

### 3.2. Cell Culture, Decorin Gene Transduction and Proliferation Assay

Human U87MG glioma cells were obtained from the American Type Culture Collection (Manassas, VA, USA). Cells were cultured in Dulbecco’s modified Eagle’s medium (DMEM) supplemented with 10% fetal bovine serum (Invitrogen, Carlsbad, CA, USA), and 1% penicillin-streptomycin (Invitrogen, Carlsbad, CA, USA) at 37 °C in 5% CO_2_ [5,8]. U87MG cells (5 × 10^5^ cells) were infected with purified dsAAV-decorin (U87MG + dsAAV-decorin group) or dsAAV-EGFP (U87MG + dsAAV-EGFP group) particles at multiplicity of infection (MOI) 10,000 for 10 min in serum-free medium in an Eppendorf tube, and then replaced by serum-containing medium and cultured in 10-cm dish for three days without replacement of the supernatant. Phosphate buffered saline (PBS) without viral particles (U87MG group) was used to treat the cells as a mock-infection control. Cell morphology was evaluated daily using a phase-contrast microscope for three days. Proliferation of U87MG cells in response to decorin gene transfer was determined by using a WST-1 cell proliferation assay kit (Roche, Indianapolis, IN, USA) according to the manufacturer’s instructions. The number of cells was determined by measuring the absorbance at 450 nm by an ELISA reader (uQuant, Bio-Tek Instrument Inc., Winooski, VT, USA) to detect the water-soluble formazan from cleavage of WST-1 by mitochondrial dehydrogenases of viable cells. After three days of transduction, cells from U87MG + dsAAV-decorin, U87MG + dsAAV-EGFP and U87MG groups were harvested for proteomic analysis.

### 3.3. Animal Study

The animal study was performed as previously described [5,8]. In the therapeutic group, U87MG cells (5 × 10^5^ cells/5 μL for each mouse) were stereotactically injected (i.c.) into the brain of nude mice. Five days post-implantation, 5 μL of dsAAV-decorin vectors were delivered via intracranial injection to the same site of the tumor. Long-term survival of the tumor-bearing mice was evaluated. In the preventive group, U87MG cells (5 × 10^5^/10 μL cells for each mouse) or the same number of glioma cells mixed with AAV vector in a volume of 10 μL were co-injected to the brain of mice.

### 3.4. Western Blot

Western blot analysis was performed on U87MG cells to examine the overexpression of the decorin protein in dsAAV-decorin transduced cells as previously described [5,8]. Briefly, following two rinses with ice-cold phosphate-buffered saline, the cells were lysed in 100 μL ice-cold RIPA solution (100 mM Tris, pH 8.0, 300 mM NaCl, 2% Nonidet P-40, 1% sodium deoxycholate, 0.2% SDS). Cell debris was removed by centrifugation at 20,000× *g* for 15 min at 4 °C. Ten μg of total proteins were separated by 10% SDS-PAGE followed by transferring the separated proteins onto a PVDF membrane. The membrane was blocked in 20 mL blocking buffer (Tris-buffered saline, pH 8.0, containing 0.05% Tween-20, TBST in 5% nonfat milk) at room temperature for 1 h, then incubated with a primary antibody at room temperature for 1 h, then washed with TBST four times, and followed by incubation with a secondary antibody in the same blocking buffer for 1 h at room temperature. The reacted proteins on the membrane were visualized using an enhanced chemiluminescence (ECL) detection kit.

### 3.5. Sample Preparation and 2-DE

The harvested cells infected by dsAAV-decorin or dsAAV-EGFP (2 × 10^5^ cells) were homogenized in 250 μL of a lysis buffer (7 M urea, 2 M thiourea, 4% CHAPS, and 0.5% IPG buffer pH 3–10) using a sonication probe. The homogenates were centrifuged at 20,000× *g* for 15 min at 20 °C to remove tissue and cell debris. Protein concentration in the supernatants was determined by a modified Bradford method [65]. Two hundred μg of total proteins for each sample were loaded onto an IPG strip (Immobiline DryStrip 3–10, GE Healthcare, Piscataway, NJ, USA) for simultaneous rehydration. Isoelectric focusing electrophoresis (IEF) was performed with the following voltage-time program: 50 V for 12 h; 500 V for 1 h; 1000 V for 1 h; 8000 V, for a total of 120,000 V/h. Immediately after being focused, IPG strips were sealed in plastic holders and stored at −20 °C. Prior to SDS-PAGE, IPG strips were equilibrated in 6 M urea/2% SDS/1% DTT/50 mM Tris (pH 8.4)/30% glycerol for 15 min, followed by equilibration in 6 M urea/2% SDS/2.5% iodoacetamide/50 mM Tris (pH 8.4)/30% glycerol for 15 min. The second dimension separation was run for 5 h using a vertical electrophoresis system (GE Healthcare, Piscataway, NJ, USA) in 1 mm 12.5% gels at 20 mA/gel at 15 °C. After electrophoresis, gels were fixed and stained using SyproRuby according to the manufacturer’s instructions (Invitrogen, Carlsbad, CA, USA), and were then scanned with Typhoon Trio laser scanners (GE Healthcare, Piscataway, NJ, USA). For the preparative gel, 500 μg of protein was loaded and the gel was stained with Coomassie Brilliant Blue G-250 to visualize the protein spots for excision. The spots were digested using trypsin and subjected to direct MS protein identification.

### 3.6. Spot Detection, Quantification, and Comparisons

2-D gel analysis software (ImageMaster 2D platinum, GE Healthcare, Piscataway, NJ, USA) was used in this study for spot detection, gel matching, and spot quantification. The student *t* test was applied to compare the spot relative volume (%vol) in gels derived from cells of U87MG + dsAAV-decorin, U87MG + dsAAV-EGFP and U87MG groups. Significant spots that showed at least 1.5-fold difference in relative volume between the groups were selected for protein identification.

### 3.7. Digestion of Proteins

All of the protein spots of interest were manually excised. The gel pieces were destained in 60% acetonitrile in 25 mM ammonium bicarbonate buffer, pH 8.5, and dehydrated with 100% acetonitrile. The shrunken gel pieces were re-swollen in 25 mM ammonium bicarbonate buffer, dehydrated again in 100% acetonitrile, and dried in a SpeedVac (Thermo Scientific, Milford, MA, USA). The gel pieces were re-hydrated in 10 μL trypsin solution (20 μg/mL) for 1 h, followed by addition of 5 μL 25 mM ammonium bicarbonate buffer to completely immerse the gel pieces. After incubation overnight at 37 °C, 0.5 μL incubation buffer was mixed with 0.5 μL matrix solution (CHCA 2 mg/mL in 50% acetonitrile and 1% TFA) and pipetted directly onto the stainless steel sample plate of the mass spectrometer. The samples were analyzed by MALDI-TOF MS (Biflex IV, Bruker Daltonics, Bremen, Germany). In cases where the MS signals were weak, the peptides were enriched by C18 ZipTip (Millipore, Billerica, MA, USA) according to the manufacturer’s instructions. The bound peptides were eluted from the ZipTip using 1 μL CHCA, which was directly deposited onto the metal plate.

### 3.8. MALDI-TOF MS

The mass spectrometer utilized for protein analysis was a Bruker Biflex IV MADLI-TOF MS (Bruker Daltonics, Bremen, Germany). For peptide mass fingerprint (PMF) each mass spectrum was averaged from signals generated from 500 laser shots. The mass spectra were processed using Flexanalysis™ and Biotools™ softwares (Bruker Daltonics, Bremen, Germany) and the data were searched against UniProt database (http://www.pir.uniprot.org, The Universal Protein Resource (UniProt)) by a MS-Fit database-mining engine [66,67]. For each PMF search, the mass tolerance was set to 100 ppm. One missed tryptic cleavage was allowed.

### 3.9. Statistics

The data are presented as the mean ± SD. Student’s *t* test was used for the statistical analysis. Differences were considered significant at *p* < 0.05.

## 4. Conclusions

**Figure 7.**
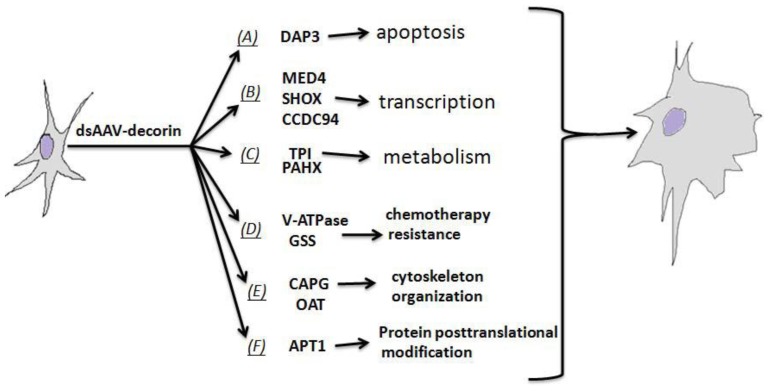
Schematic diagram of the multiple biochemical mechanisms evoked by dsAAV-decorin in U87MG cells. Potential effects of dsAAV-decorin on apoptosis (**A**); transcription (**B**); metabolism of glucose and lipid (**C**); chemotherapy resistance (**D**); cytoskeleton organization (**E**); and protein posttranslational modification (**F**) were summarized based on published literature.

In summary, using dsAAV-decorin for stable and high-level decorin expression, we demonstrated that decorin significantly inhibited malignant U87MG glioma growth in the brain of animals and altered the expressions of several proteins that are important in cell apoptosis, gene transcription, chemotherapy resistance of cancer cells, mitosis, and fatty acid metabolism and modification (Figure 7). Our proteomics data suggest that dsAAV-decorin induces differentiation of glioma cells by evoking multiple biochemical mechanisms that render human glioma cells vulnerable to chemical or radiation therapies. These data may shed light on the anti-glioblastoma mechanisms of decorin described in this report and previous studies [21,23,68], and suggest that dsAAV-decorin could be a potential candidate for gene therapy in patients with malignant glioma, and as an adjuvant for other modalities of glioma therapy.

## Figures and Tables

**Table 1.** Identified proteins. The spot number, MOWSE score, UniProt database accession number, and protein name abbreviation are shown, followed by the theoretical and observed molecular mass (*Mr*) and isoelectric point (p*I*). The number of matching peptides and sequence coverage were calculated using Biotools™ software. The direction of the change in protein expression is also indicated.

Spot No.	MOWSE score	Accession No.	Protein name abbreviation	Protein name	Theoretical *Mr*(kDa)/p*I*	Observed *Mr* (kDa)/p*I*	Matching peptides	Sequence coverage (%)	Change direction
1	18471	P51398	DAP3	Death associated protein 3	45.5/9.0	49/5.9	8	20.4	up
2	1384	Q9NPJ6	MED4	Mediator of RNA polymerase II transcription subunit 4	29.7/5.0	34/7.2	5	23.7	up
3	2803	P60174	TPI	Triosephosphate isomerase	26.5/6.5	25/7.4	5	26.6	down
4	249	Q5HYX6	SHOX	Short stature homeobox	25.5/6.3	20/7.4	4	17.3	up
5	265502	P04264	KRT1	Keratin, type II cytoskeletal 1	65.8/8.2	62/6.1	14	33	down
6	11999	P21281	V-ATPase	V-type proton ATPase subunit B, brain isoform	56.5/5.6	54/6.1	9	18.4	down
7	5656	P48637	GSS	Glutathione synthetase	52.3/5.7	50/6.1	10	25.7	down
8	25712	P04181	OAT	Ornithine aminotransferase, mitochondrial	48.5/6.6	45/6.7	9	25.1	down
9	4120	P40121	CAPG	Macrophage-capping protein	38.5/5.9	39/6.4	8	30.2	down
10	111	O14832	PAHX	Phytanoyl-CoA dioxygenase, peroxisomal	38.5/8.7	35/6.1	7	19.2	down
11	360	O75608	APT1	Acyl-protein thioesterase 1	24.6/6.3	26/6.5	6	28.7	down
12	266	Q9BW85	CCDC94	Coiled-coil domain-containing protein 94	37/5.8	35/5.9	4	21.4	up
